# Comparative field evaluation of kelambu traps, barrier screens and barrier screens with eaves for longitudinal surveillance of adult *Anopheles* mosquitoes in Sulawesi, Indonesia

**DOI:** 10.1186/s13071-019-3649-7

**Published:** 2019-08-13

**Authors:** Jenna R. Davidson, Isra Wahid, Rusdiyah Sudirman, Victoria Makuru, Hajar Hasan, Andi Muhammad Arfah, Nirwana Nur, Muhammad Yusuf Hidayat, Allison L. Hendershot, Honglin Xiao, Xiaoyu Yu, Puji Budi Setia Asih, Din Syafruddin, Neil F. Lobo

**Affiliations:** 1 Eck Institute for Global Health, University of Notre Dame, Notre Dame, IN 46556 USA; 2 Department of Parasitology, Faculty of Medicine, Universitas Hasanuddin, Makassar, 90245 Indonesia; 3 Eijkman Institute for Molecular Biology, Jakarta, Indonesia

**Keywords:** Kelambu trap, Barrier screens, Barrier screens with eaves, Bionomics

## Abstract

**Background:**

Sampling methodologies for mosquitoes that are capable of transmitting vector-borne infectious diseases provide critical information on entomological endpoints. Reliable and meaningful field data is vital to the understanding of basic vector biology as well as disease transmission. Various traps take advantage of different vector behaviors and are inevitably subject to sampling biases. This study represents the first comparison of kelambu traps (KT) to barrier screens (BS), barrier screens with eaves (BSE) and indoor and outdoor human landing catches (HLCs).

**Methods:**

Two trap comparison studies were undertaken. In the first study, mosquitoes were collected in Karama over 26 trapping nights to evaluate the kelambu trap relative to indoor and outdoor HLCs. In the second study, mosquitoes were collected in Karama over 12 trapping nights to compare the kelambu trap, barrier screen, barrier screen with eaves and outdoor HLCs. The kelambu trap, barrier screen and barrier screen with eaves obstruct the flight of mosquitos. HLCs target host-seeking behaviors.

**Results:**

There was no significant difference between indoor and outdoor HLCs for overall *Anopheles* mosquito abundance. All five of the molecularly identified *Anopheles* species collected by HLCs, *An. aconitus*, *An. barbirostris*, *An. peditaeniatus*, *An. vagus* and *An. tessellatus*, are reported as vectors of malaria in Indonesia. The kelambu trap (*n* = 2736) collected significantly more *Anopheles* mosquitoes than indoor HLCs (*n* = 1286; *Z* = 3.193, *P* = 0.004), but not the outdoor HLCs (*n* = 1580; *Z* = 2.325, *P* = 0.053). All traps collected statistically similar abundances for the primary species, *An. barbirostris*. However, both comparison studies found significantly higher abundances for the kelambu trap for several secondary species compared to all other traps: *An. nigerriumus*, *An. parangensis*, *An. tessellatus* and *An. vagus*. The kelambu trap retained the highest species richness and Gini-Simpson’s diversity index for both comparison studies.

**Conclusions:**

This study demonstrates that the kelambu trap collects overall *Anopheles* abundance and species-specific abundances at statistically similar or higher rates than HLCs in Sulawesi, Indonesia. Therefore, the kelambu trap should be considered as an exposure-free alternative to HLCs for research questions regarding *Anopheles* species in this malaria endemic region.

## Background

Sampling methodologies for mosquitoes that are capable of transmitting vector-borne infectious diseases provide critical information on several entomological endpoints including species present, temporal population densities and distributions and bionomic characteristics, as well as the effects of control measures on populations. Reliable and meaningful field data is vital to the understanding of basic vector biology as well as disease transmission. Various traps take advantage of different vector behaviors and are therefore subject to sampling biases. For example, host-baited traps target female, host-seeking mosquitoes, as they are attracted to their hosts based on the odors they emit [1], whereas artificial resting collections such as pit traps, resting boxes and wood-fiber pots take advantage of mosquito resting behaviors [2–6]. Research shows that vector behaviors can vary within small geographical scales [7] as well as in response to interventions [8]. Thus, the efficacy of sampling methodologies will vary depending on geographical location, and consequently, evaluation of these methods is important to determine their functionality in different localities.

The gold standard collection method, human landing catches (HLCs), are used for the collection of human host-seeking *Anopheles* mosquitoes and are the most indicative collection method of mosquito human-feeding activity. However, they have come under scrutiny due to ethical concerns of exposing collectors to infectious bites [9]. Although ethical concerns about malaria incidence in HLC collectors may be mitigated by two compelling studies that demonstrate no difference in infection rates in the community *versus* those conducting HLCs, as well as the positive impacts of prophylaxis [10, 11] there exists the risk of non-malarial arboviral disease transmission for which there is no prophylaxis or treatment, such as dengue [12–14]. Currently, HLCs are widely used by mosquito and disease surveillance studies in Indonesia. The continued use of the HLC sampling method as a surveillance tool in areas with extensive malaria and arboviral transmission, like Indonesia, stresses the need to develop and characterize alternative, comparable and safer sampling methodologies suitable for host-seeking mosquitoes.

Several exposure-free traps have been evaluated in comparison to HLCs in Indonesia, including CDC light traps, resting pots and boxes, malaise traps and tent traps [15, 16]. The CDC light trap has been used in West Sumba District, East Nusa Tenggara Province, Indonesia to collect 13 different species, namely *An. aconitus*, *An. annularis*, *An. barbirostris*, *An. flavirostris*, Hyrcanus group, *An. indefinitus*, *An. kochi*, *An. leucosphyrus* group, *An. maculatus* (*s.l.*), *An. subpictus* (*s.l.*), *An. sundaicus* (*s.l.*), *An. tessellatus* and *An. vagus*, at rates comparable to HLCs [15]. However, in another study that represents different localities, the CDC light trap demonstrated lower rates of capture compared to HLCs [16]. In each case, trapping efficacy was varied based on location. Furthermore, there is a general lack of published information of exposure-free trapping for Indonesia, so there is a need for replication and local translation in the geographically and biologically diverse country. Finally, successful implementation of any trap relies on local mosquito population density dynamics and species-specific mosquito behaviors, neither of which are well documented in Sulawesi.

The barrier screen (BS) was developed to determine an unbiased sample of blood-fed and host-seeking mosquitoes collected during field investigations [7, 17]. Tested in Indonesia, Solomon Islands and Papua New Guinea, the BS has been demonstrated to be an effective sampling methodology for *Anopheles*, *Culex* and *Aedes* species while being less cumbersome and more economical than many other exposure-free trapping methodologies [7, 17]. However, a limitation of the barrier screen is its unobstructed top, which may allow intercepted mosquitoes to crawl or fly over the trap before collections take place, thereby reducing the number of mosquitoes caught. In this study, sampling with BS was extended to the use of barrier screens with eave covers (BSE) to limit mosquitoes from crawling or flying over the trap. The eaves were designed to increase the catching efficacy of the BS.

The kelambu trap (kelambu translates to “mosquito net”) developed for this study is a modified bednet trap that is used to intercept free-flying mosquitoes while defining flight patterns. It is square and divided along both axes into four quadrants, enabling potential vectors to be intercepted from four directions. The kelambu trap is devised to make mosquito entrance to the trap easy and exit difficult.

This study represents the first comparison of the KT, BS and BSE (outdoor net-based interception traps) to outdoor HLCs. Hereafter, “net traps” will be used to refer to KT, BSE and BS as a group. The primary aims of the study were to (i) compare outdoor and indoor HLCs to determine differences, if any, between mosquito abundance and species compositions in Karama for indoor and outdoor populations; (ii) evaluate the efficacy of the KT by comparing mosquito abundance, species composition and flight activity to indoor and outdoor HLCs; and (iii) evaluate the efficacy of the KT by comparing mosquito abundance, species composition, abdominal status and flight activity to the outdoor HLCs, BS and BSE. The information generated from this study will aid researchers in choosing the appropriate outdoor sampling methodologies for surveys of mosquitoes as well as provide more options for exposure-free sampling methodologies for entomological investigations.

## Methods

### Site description

**Fig. 1:**
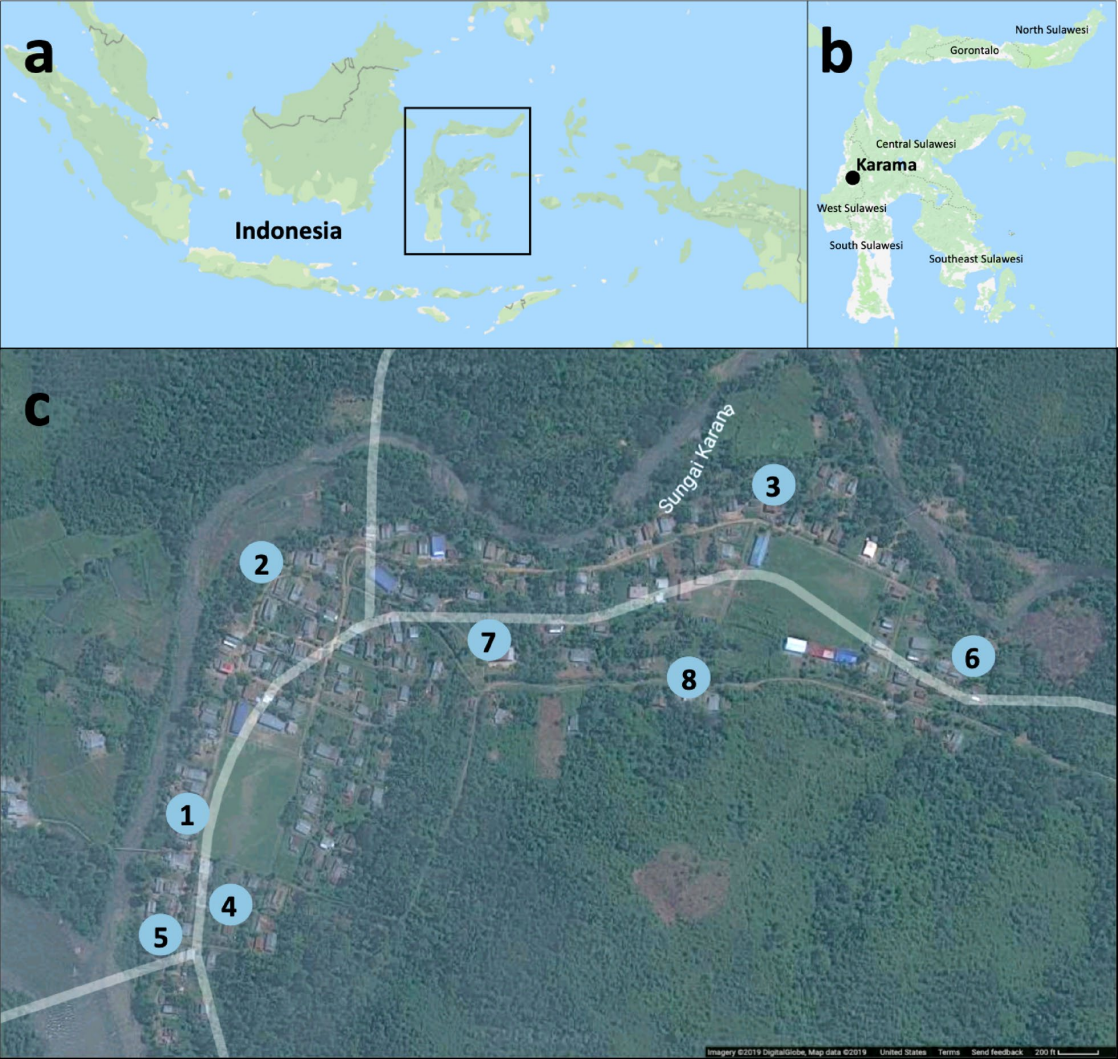
Map of Karama field collection sites. Mosquitoes were collected using kelambu traps, barrier screens, barrier screens with eaves and human landing catches inside and outside at eight sites. Sites were located both along the river Karama edge and at the borders of the nearby forest. The map was created using Google: Imagery 2019 DigitalGlobe, Map data 2019

Karama, Indonesia is a village in the northwestern regency of Mamuju, West Sulawesi (Fig. 1), and has an area of 1 km^2^. This isolated village, bordered by the Karama River, is partly located in the flood plain with areas reaching into the foothills and surrounded by forest. The main economic activity in the region is agriculture, with the primary crop being rice. Houses in this area are made of wood or concrete with thatched roofs. Low-lying houses are elevated with stilts because of consistent flooding in the area. The open construction of these primarily wooden houses allows for free mosquito entry from all directions. This remote area has stable, year-round malaria transmission with increased incidences during the rainy season (November to March) (Dr Isra Wahid, personal communication) [5].

### Trap descriptions

#### Kelambu trap (KT)

**Fig. 2:**
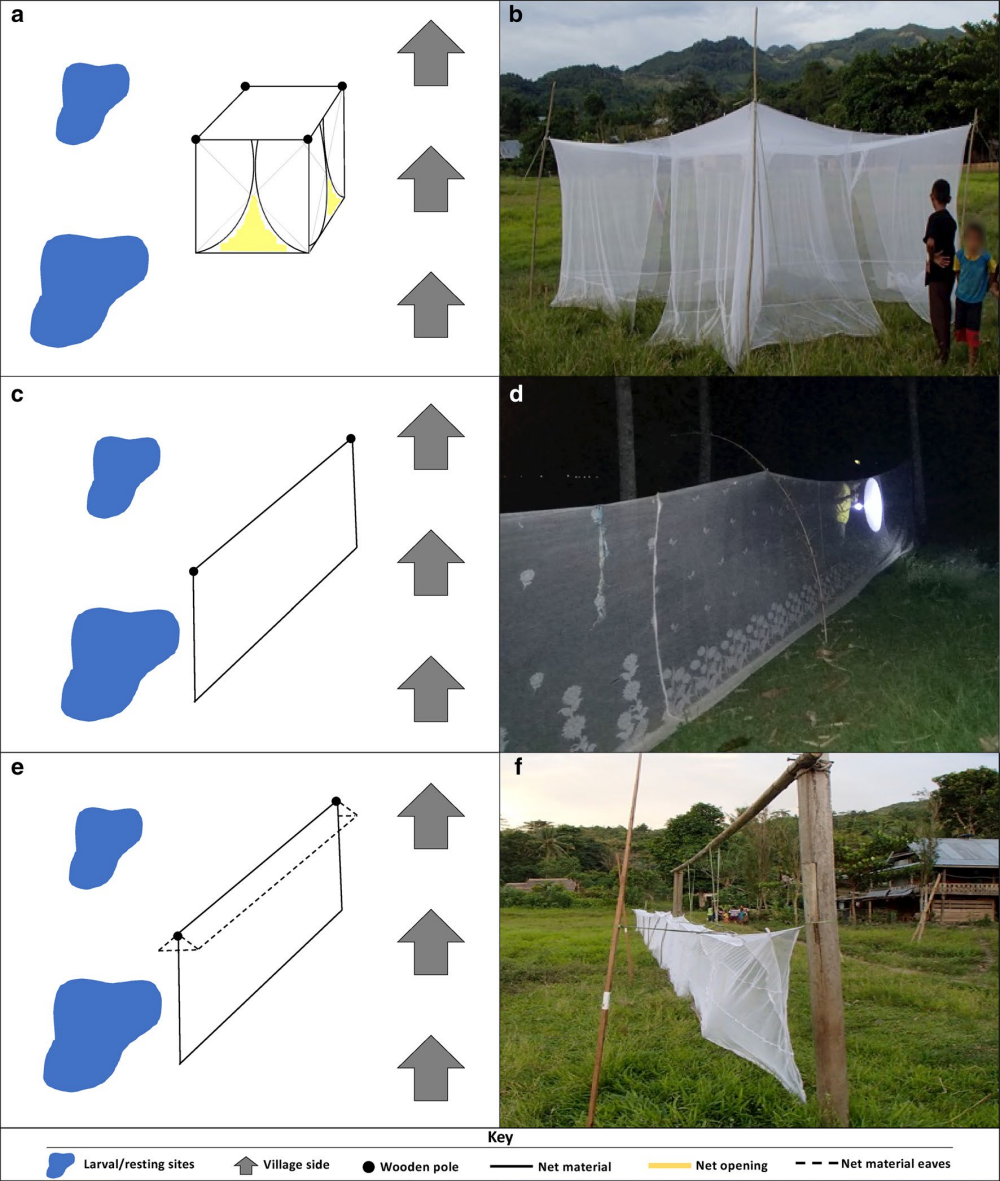
Net sampling methodologies. **a** Kelambu trap schematic. **b** Kelambu trap photo. **c** Barrier screen schematic. **d** Barrier screens photo. **e** Barrier screen with eaves schematic. **f** Barrier screen with eaves photo

The KT is an attractant-free, modified bednet trap that targets free-flying mosquitoes (Fig. 2a, b). The trap is separated orthogonally from each corner along the axes to give four triangular quadrants, each of which is partially open to allow for mosquito entry and the determination of mosquito flight direction. The KT is devised to make mosquito entrance to the trap easy and exit difficult. Mosquitoes were collected from each quadrant by aspiration for 10 min every hour from 18:00 to 06:00 h. Location, time of collection, abdominal status and flight direction (determined by whether mosquitoes were collected on the village side or the larval/resting site side of the KT) was recorded for each mosquito.

#### Human landing catches (HLCs)

Informed consent was obtained and HLCs were conducted as described by Gimnig et al. [10]. HLCs were performed between 18:00 and 06:00 h. Paired collections were performed inside and outside sentinel houses. Collections were done in 2-h shifts, with a single collector indoors and a single collector outdoors for each house (*n* = 8). After each 2-h period, the two collectors swapped positions to reduce collector bias. Location and time of collection was recorded for all mosquitoes.

#### *Barrier screen* (BS)

The BS was constructed with 2 m high untreated bednet material secured to wooden poles at 2 m intervals for a length of 10 m (Fig. 2c, d). The BS was set up and located as previously described [17]. The BS was examined for mosquitoes every hour between 18:00 and 06:00 h. Two collectors walked along each side of the trap for 15–20 min every hour, using a flashlight to spot and mouth aspirator to collect resting mosquitoes. Time of collection and flight direction (determined by whether mosquitoes were collected on the village side or the larval/resting site side of the BS) was recorded for mosquitoes.

#### Barrier screen with eaves (BSE)

The BSE was constructed in the same manner as the BS, but with 20 cm untreated bednet material eaves at the top to prevent mosquitoes from escaping over the vertical netting (Fig. 2e, f). Mosquitoes were collected from the BSE and information was recorded in the manner described for the BS.

### Trap comparison Study 1: evaluation of KT compared to indoor HLCs and outdoor HLCs

Mosquitoes were collected in Karama over 26 trapping nights from April 2013 to March 2015 (Table 1) to evaluate the KT relative to indoor and outdoor HLCs. Collections encompassed both the dry and wet season. Eight collection sites were used for this comparison (Fig. 1). This study addressed aims to (i) compare outdoor and indoor HLCs to determine differences, if any, between mosquito abundance and species compositions in Karama for indoor and outdoor populations; and (ii) evaluate the efficacy of the KT by comparing mosquito abundance, species composition and flight activity to indoor and outdoor HLCs.

**Table 1:** Overview of mosquito collection nights by date. Four collection methods, KT, BS, BSE, and HLCs, were utilized in Karama, Indonesia between 2013 and 2015

Trapping method	April-May 2013 (no. of nights)	September 2013 (no. of nights)	December 2013 (no. of nights)	May 2014 (no. of nights)	January 2015 (no. of nights)	March 2015 (no. of nights)	Total no. of collection nights
KT	9	7	3	2	2	3	26
HLC	9	7	3	2	2	3	26
BS	9	–	–	–	–	3	12
BSE	9	–	–	–	–	3	12

*Abbreviations*: KT, kelambu trap; BS, barrier screens; BSE, barrier screens with eaves; HLC, human landing catches

### Trap comparison Study 2: evaluation of KT compared to BS, BSE and outdoor HLCs

Mosquitoes were collected in Karama over 12 trapping nights from April 2013 to March 2015 (Table 1) to evaluate the KT relative to the BS, BSE and outdoor HLCs. Collections encompassed both the dry and wet season. Eight collection sites were used for this comparison (Fig. 1). All 12 of the trapping nights of trap comparison study 2 were also used in trap comparison study 1. Therefore, data for these 12 nights for the KT and outdoor HLCs are used in both studies. This addressed aim (iii) evaluate the efficacy of the KT by comparing mosquito abundance, species composition, abdominal status and flight activity to the outdoor HLCs, BS and BSE.

### Site rotation and design

Sentinel houses (×8) at each collection site were used for indoor and outdoor HLCs (Fig. 1). Net traps were positioned outside, near each sentinel house. All traps were randomly rotated between sites with only one trap being used at a site on a given night. Additionally, some nights in the study had multiple collectors for both indoor and outdoor HLCs; therefore, HLC abundance was calculated as per person (divided by the number of collectors each night).

### Abundance

For all comparisons in both studies, *Anopheles* abundance was examined and calculated as mean nightly abundance for the KT, BS, BSE and HLCs at each collection site.

### Species identification

*Anopheles* sampled from all traps were morphologically identified in the field to species [18].

Molecular identification was performed on *Anopheles* mosquitoes in which approximately 10% of mosquitoes from each trap type were randomly selected and molecularly identified using internal transcribed spacer region II and cytochrome oxidase I loci [19, 20].

### Data analysis

All statistical analyses were completed in R v.3.5.2 [21]. Catches were analyzed using generalized linear models (GLMs; R package *MASS* [22]) with negative binomial distributions, followed by *post-hoc* Tukey comparisons between collection methods (R package *multcomp* [23]). Finally, differences in mean night mosquito abundances for specific species, morphologically identified, were analyzed to investigate trap-specific biases. Species with smaller overall abundances (*n* < 50) were not analyzed for statistically significant mean nightly abundances. Statistical analyses were not performed on molecularly identified specimens as only a small subset of randomly selected mosquitoes were molecularly identified.

The Gini-Simpsonʼs diversity index (1 − λ) was used to measure trap ability to sample the diversity of mosquitoes in Karama, Indonesia. A higher value indicates more diversity of species captured by a trap, technically being a percentage chance that two mosquitoes chosen at random within the trap would be different species. Therefore, a value of 1 is impossibly high unless there is only 1 species present at the site. The index accounts for numerical variance towards dominant species as well as species known to be at the site, as determined by other trapping methods, but not captured by the trap in question [24]. Gini-Simpson’s diversity index was calculated as:

$$1 - \lambda = 1 - \mathop \sum \limits_{i = 1}^{R} p_{i}^{2} = 1 - \frac{1}{{{}_{ }^{2} D}}$$

where *R* is species richness (total number of species present) and *p* is the weighted arithmetic mean of the proportional abundances [25, 26].

## Results

### Trap comparison Study 1: evaluation of KT compared to indoor HLCs and outdoor HLCs

To evaluate the efficacy of the KT to indoor HLCs and outdoor HLCs, mosquitoes were collected for 26 nights.

#### Overall abundance

Over the 26 collection nights there was a significant difference in the *Anopheles* abundance between the KT (*n* = 2736; mean per night, 105.2 ± 17.53), indoor HLCs (*n* = 1286; mean per night 49.46 ± 8.30) and outdoor HLCs (*n* = 1580; mean per night, 60.77 ± 10.17) (*F*_(2, 75)_ = 11.323, *P* = 0.003). However, *post-hoc* Tukey comparisons revealed no statistical significance for *Anopheles* abundance between only indoor HLCs and outdoor HLCs (*Z* = 0.869, *P* = 0.660). Meanwhile, the KT collected significantly more *Anopheles* mosquitoes than the indoor HLCs (*Z* = 3.193, *P* = 0.004), but not the outdoor HLCs (*Z* = 2.325, *P* = 0.053).

#### Species composition

To evaluate species compositions, morphological identification was performed on all female *Anopheles* mosquitoes collected during the 26 collection nights (*n* = 5602). Mosquitoes were morphologically identified to 15 different species (Table 2). For the primary species, *An. barbirostris*, there were no statistical differences in abundance between traps (Table 3). The kelambu trap collected statistically higher abundances of *An. nigerrimus*, *An. parangensis*, *An. tessellatus* and *An. vagus* than both indoor and outdoor HLCs (Table 3). Indoor and outdoor HLCs collected statistically similar abundances for all species except *An. vagus*, for which the outdoor HLCs collected more (Table 3). There were no statistical differences in abundance between traps for *An. barbumbrosus* or *An. umbrosus* (Table 3). Abundance values for *An. aconitus*, *An. flavirostris*, *An. hyrcanus*, *An. indefinitus*, *An. kochi*, *An. maculatus*, *An. schuefneri* and *An. sulawesi* were too low to statistically analyze (Table 2).

**Table 2:** Species identified morphologically for trap comparison Study 1

Species	KT	Indoor HLC	Outdoor HLC
*An. aconitus*	17	1	3
*An. barbirostris*	1649	1111	1330
*An. barbumbrosus*	49	23	24
*An. flavirostris*	5	1	1
*An. hyrcanus*	10	1	2
*An. indefinitus*	5	1	1
*An. kochi*	3	0	0
*An. maculatus*	5	1	2
*An. nigerrimus*	320	53	61
*An. parangensis*	285	32	44
*An. schueffneri*	1	0	0
*An. sulawesi*	0	1	0
*An. tessellatus*	90	3	3
*An. umbrosus*	35	54	77
*An. vagus*	262	4	32

*Abbreviations*: KT, kelambu trap; HLC, human landing catches

**Table 3:** Species abundance comparison between trap type for trap comparison studies 1 and 2

Species	Trap comparison	*Z*-value	*P*-value
Trap comparison Study 1			
*An. barbirostris*	KT *vs* indoor HLC	0.974	1.000
	KT *vs* outdoor HLC	0.530	1.000
	Outdoor HLC *vs* indoor HLC	0.443	1.000
*An. barbumbrosus*	KT *vs* indoor HLC	1.588	0.9918
	KT *vs* outdoor HLC	1.505	0.9958
	Outdoor HLC *vs* indoor HLC	0.085	1.000
*An. nigerrimus*	KT *vs* indoor HLC	4.181	< 0.01*
	KT *vs* outdoor HLC	3.880	0.0151
	Outdoor HLC *vs* indoor HLC	0.316	1.000
*An. parangensis*	KT *vs* indoor HLC	4.918	< 0.01*
	KT *vs* outdoor HLC	4.295	< 0.01*
	Outdoor HLC *vs* indoor HLC	0.684	1.000
*An. tessellatus*	KT *vs* indoor HLC	4.775	< 0.01*
	KT *vs* outdoor HLC	4.775	< 0.01*
	Outdoor HLC *vs* indoor HLC	0.000	1.000
*An. umbrosus*	Indoor HLC *vs* KT	0.946	1.000
	Outdoor HLC *vs* KT	1.280	0.406
	Outdoor HLC *vs* indoor HLC	0.805	1.000
*An. vagus*	KT *vs* indoor HLC	5.79	< 0.0001***
	KT *vs* outdoor HLC	3.589	0.0009***
	Outdoor HLC *vs* indoor HLC	2.709	0.0183*
Trap comparison Study 2			
*An. barbirostris*	KT *vs* BS	1.309	1.000
	KT *vs* BSE	0.870	1.000
	Outdoor HLC *vs* KT	0.029	1.000
	BSE *vs* BS	0.440	1.000
	Outdoor HLC *vs* BS	1.338	1.000
	Outdoor HLC *vs* BSE	0.899	1.000
*An. barbumbrosus*	KT *vs* BS	1.745	0.995
	KT *vs* BSE	0.616	1.000
	KT *vs* outdoor HLC	0.901	1.000
	BSE *vs* BS	1.151	1.000
	Outdoor HLC *vs* BS	0.871	1.000
	BSE *vs* outdoor HLC	0.287	1.000
*An. nigerrimus*	KT *vs* BS	3.925	0.0214*
	KT *vs* BSE	2.732	0.0323*
	KT *vs* outdoor HLC	2.675	0.0374*
	BSE *vs* BS	0.571	1.000
	Outdoor HLC *vs* BS	0.641	1.000
	Outdoor HLC vs BSE	0.058	1.000
*An. parangensis*	KT *vs* BS	4.429	< 0.001***
	KT *vs* BSE	3.120	0.009**
	KT *vs* outdoor HLC	6.124	< 0.001***
	BSE *vs* BS	1.377	0.512
	BS *vs* outdoor HLC	2.156	0.134
	BSE *vs* outdoor HLC	3.419	0.0034**
*An. tessellatus*	KT *vs* BS	3.017	0.0128*
	KT *vs* BSE	2.771	0.0272*
	KT *vs* outdoor HLC	3.560	0.0019**
	BSE *vs* BS	0.294	0.991
	BS *vs* outdoor HLC	1.529	0.410
	BSE *vs* outdoor HLC	1.744	0.292
*An. umbrosus*	KT *vs* BS	2.570	0.049*
	KT *vs* BSE	0.332	0.987
	Outdoor HLC *vs* KT	1.776	0.282
	BSE *vs* BS	2.277	0.102
	Outdoor HLC *vs* BS	4.069	< 0.001***
	Outdoor HLC *vs* BSE	2.099	0.151
*An. vagus*	KT *vs* BS	3.985	< 0.001***
	KT *vs* BSE	2.630	0.0429*
	KT *vs* outdoor HLC	3.751	0.001**
	BSE *vs* BS	1.420	0.586
	Outdoor HLC *vs* BS	0.255	0.994
	BSE *vs* outdoor HLC	1.169	0.646

*P ≤ 0.05, **P ≤ 0.01, *** *P* ≤ 0.001

*Abbreviations*: KT, kelambu trap; BS, barrier screens; BSE, barrier screens with eaves; HLC, human landing catches

#### Species diversity

The KT had the greatest species richness (*R* = 14) and Gini-Simpsonʼs diversity index (1 − λ = 0.6014). Outdoor HLCs had the lowest species richness (*R* = 12) but the second highest Gini-Simpsonʼs diversity index (1 − λ = 0.2861). Indoor HLCs had middle species richness (*R* = 13) and the lowest Gini-Simpsonʼs diversity index (1 − λ = 0.2492).

#### Molecular identification

To evaluate species composition, molecular identification was performed on *Anopheles* collected with KTs, indoor HLCs and outdoor HLCs for the 26 collection nights. Of the molecularly identified *Anopheles* mosquitoes, KTs (*n* = 546) collected 60.8% *An. barbirostris*, 24.5% *An. vagus*, 8.8% *An. peditaeniatus*, 4.0% *An. tessellatus*, 1.6% *An. aconitus* and 0.2% *An. karwari.* Outdoor HLCs (*n* = 470) collected 96.0% *An. barbirostris*, 1.7% *An. peditaeniatus*, 1.7% *An. vagus*, 0.4% *An. tessellatus* and 0.2% *An. aconitus*. Outdoor HLCs did not collect any *An. karwari*. Indoor HLCs (*n* = 530) collected 96.2% *An. barbirostris*, 1.9% *An. peditaeniatus*, 1.7% *An. vagus* and 0.2% *An. tessellatus*.

#### Flight activity

**Fig. 3:**
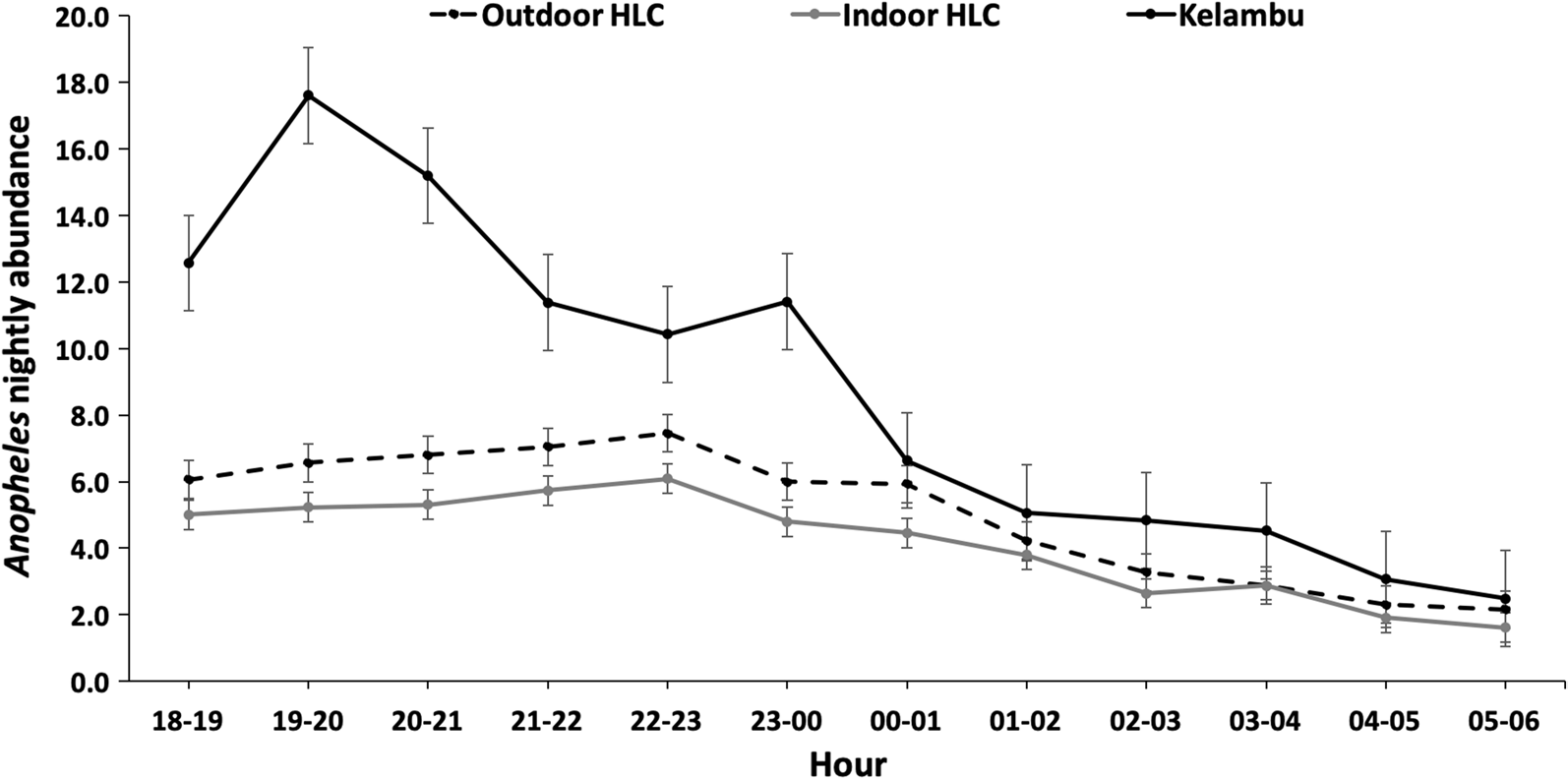
Comparison of the *Anopheles* nightly abundance collected with kelambu traps, outdoor HLCs and indoor HLCs by hour

To evaluate activity, the mean *Anopheles* abundance was examined by collection time for KTs, indoor HLCs and outdoor HLCs. For KTs, the highest activity was seen from 19:00 to 20:00 h and decreased throughout the night. Activity for both indoor and outdoor HLCs gradually rose until 22:00–23:00 h then decreased throughout the night (Fig. 3). Mosquito activity for indoor and outdoor HLCs mirrored each other throughout the night (Fig. 3).

### Trap comparison Study 2: evaluation of KT compared to BS, BSE and HLCs

To evaluate the efficacy of the KT to BS, BSE and HLCs, mosquitoes were collected over 12 nights. All 12 of the trapping nights of trap comparison study 2 were also used in trap comparison study 1; therefore, data for these 12 nights for the KT and outdoor HLCs are used in both studies. Only outdoor HLCs were used for HLC data to streamline comparison between HLCs and net traps. Streamlining the comparison of HLCs to net traps by eliminating indoor HLC data was justified by the reasoning that indoor and outdoor HLCs were statistically similar in trap comparison study 1 except that outdoor HLCs collected a statistically higher abundance for *An. vagus*.

#### Overall abundance

Throughout the 12 collection nights there was a significant difference in *Anopheles* abundance between the KT (*n* = 1898; mean per night, 158.2 ± 36.64), BSE (*n* = 886; mean per night, 73.83 ± 17.20), BS (*n* = 659; mean per night, 54.92 ± 12.84) and outdoor HLCs (*n* = 1172; mean per night, 97.83 ± 22.73) (*F*_(3, 44)_ = 11.495, *P* = 0.009). However, *post-hoc* Tukey comparisons revealed the only statistical difference between two specific traps was the comparison between the KT and BS (*Z* = 3.214, *P* = 0.007).

#### Species composition

To evaluate species composition, morphological identification was performed on *Anopheles* collected with the KT, BSE, BS and HLCs for the 12 collection nights (*n* = 4615). Mosquitoes were identified to 14 different species (Table 4). For the primary species, *An. barbirostris*, there was no statistical difference in abundance between trap types (Table 3). The kelambu trap collected statistically higher abundances for *An. nigerrimus*, *An. parangensis*, *An. tessellatus* and *An. vagus* than all other traps types (Table 3). The barrier screen with eaves also caught a statistically higher abundance of *An. parangensis* than HLCs (Table 3). For *An. umbrosus*, both the KT and HLCs caught a statistically higher abundance than the BS (Table 3). There were no statistical differences in abundance between traps for *An. barbumbrosus* (Table 3). Abundance values for *An. aconitus*, *An. flavirostris*, *An. hyrcanus*, *An. indefinitus*, *An. kochi*, *An. maculatus* and *An. subpictus* were too low to statistically analyze (Table 4).

**Table 4:** Species identified morphologically for trap comparison Study 2

Species	KT	BS	BSE	Outdoor HLC
*An. aconitus*	15	4	8	0
*An. barbirostris*	964	525	644	977
*An. barbumbrosus*	28	10	20	17
*An. flavirostris*	2	0	0	1
*An. hyrcanus*	3	1	0	1
*An. indefinitus*	4	2	1	1
*An. kochi*	0	1	0	0
*An. maculatus*	3	0	0	0
*An. nigerrimus*	287	42	56	58
*An. parangensis*	255	35	66	11
*An. subpictus*	0	1	0	0
*An. tessellatus*	77	7	9	1
*An. umbrosus*	31	6	26	76
*An. vagus*	229	25	56	29

*Abbreviations*: KT, kelambu trap; BS, barrier screens; BSE, barrier screens with eaves; HLC, human landing catches

#### Species diversity

The KT and BS had the highest species richness (*R* = 12), followed by HLC (*R* = 10) and BSE (*R* = 9). The KT had the highest Gini-Simpsonʼs diversity index (1 − λ = 0.684), followed by the BSE (1 − λ = 0.457), BS (1 − λ = 0.3565) and HLCs (1 − λ = 0.298).

#### Molecular identification

To evaluate species composition, molecular identification was performed on *Anopheles* collected with the KT, BS, BSE and HLCs for the 12 collection nights. Of the molecularly identified mosquitoes, KT (*n* = 233) collected 76.4% *An. barbirostris*, 10.7% *An. vagus*, 8.2% *An. peditaeniatus*, 3.0% *An. tessellatus* and 1.7% *An. aconitus*. BSE (*n* = 126) collected 60.3% *An. barbirostris*, 29.4% *An. vagus*, 5.6% *An. peditaeniatus*, 4.0% *An. tessellatus* and 0.8% *An. aconitus*. BS (*n* = 83) collected 62.7% *An. barbirostris*, 31.3% *An. vagus*, 4.8% *An. peditaeniatus* and 1.2% *An. aconitus*. Finally, outdoor HLCs (*n* = 76) collected 86.8% *An. barbirostris*, 2.6% *An. peditaeniatus*, 7.9% *An. vagus* and 1.3% each of *An. tessellatus* and *An. aconitus*.

#### Abdominal status

There was no significant difference in abundance of blood-fed mosquitoes caught between the net traps (*F*_(2, 33)_ = 3.814, *P* = 0.149) (Table 5).

**Table 5:** Abdominal status for *Anopheles* mosquitoes by collection method

Trapping method	Fed (%)	Unfed (%)	Gravid (%)	Half-gravid (%)	Male (%)	Total
BS	23 (3.9)	558 (94.6)	2 (0.3)	4 (0.7)	3 (0.5)	590
BSE	28 (3.9)	669 (94.2)	0 (0)	2 (0.3)	11 (1.5)	710
KT	32 (3.0)	1009 (95.6)	1 (0.1)	5 (0.5)	8 (0.8)	1055
Total	83 (3.5)	2236 (94.9)	3 (0.1)	11 (0.5)	22 (0.9)	2355

*Note*: Percentages were calculated for abdominal status within each trap

*Abbreviations*: BS, barrier screens; BSE, barrier screens with eaves; KT, kelambu trap

#### Flight activity

**Fig. 4:**
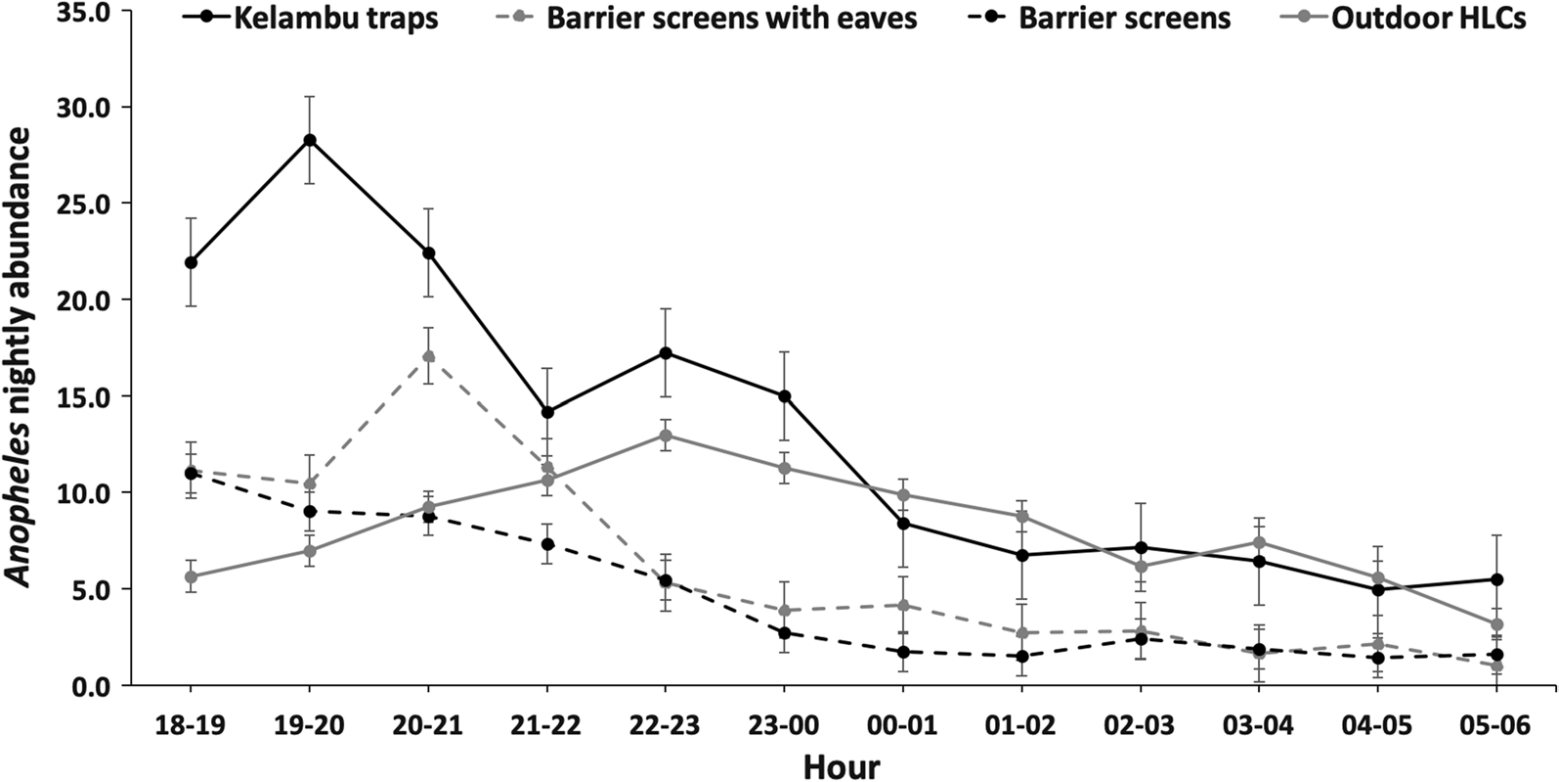
Comparison of *Anopheles* nightly abundance collected with kelambu traps, barrier screens with eaves, barrier screens and outdoor HLCs by hour. Outdoor HLCs were used for comparison to net sampling traps as trap comparison Study 1 showed no significant difference for indoor and outdoor HLCs

To evaluate mosquito activity, nightly abundance was examined by collection time for the KT, BSE, BS and outdoor HLCs. All net traps had highest activity within the first 3 h of collections, while HLC activity gradually increased until 22:00–23:00 h then gradually decreased for the remainder of the evening (Fig. 4).

**Fig. 5:**
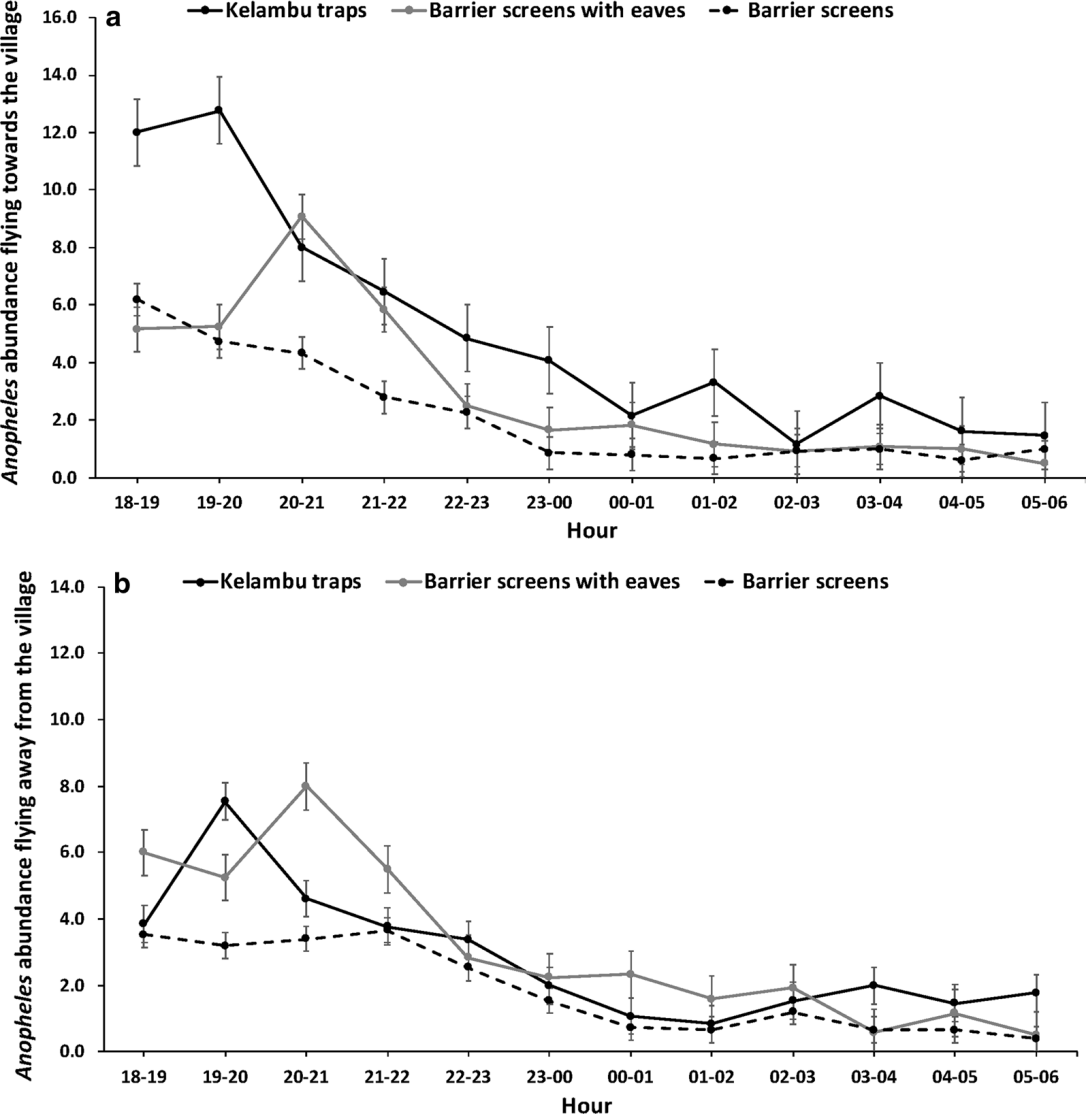
Comparison of flight direction for *Anopheles* abundance as determined by net sampling devices. **a** *Anopheles* flying towards the village by hour. **b** *Anopheles* flying away from the village by hour

Flight direction (flying towards or away from) the village was examined for the KT, BS and BSE. For *Anopheles* flying towards the village, all traps recorded highest mosquito activity in the early evening (Fig. 5a). For *Anopheles* mosquitoes flying away from the village, the highest activity also occurred in the early evening for each trap type (Fig. 5b). Activity both towards and away from the village directly mirrored overall activity.

## Discussion

Developing novel, efficacious and safe sampling methodologies that monitor mosquito vector populations is valuable for understanding entomological and epidemiological outcomes. The KT, BS, BSE and HLCs were compared in this study in Sulawesi, Indonesia.

Indoor and outdoor HLCs were compared to elucidate differences in species’ biting location preferences, which could be used to inform downstream interventions and collections. Indoor and outdoor HLCs performed statistically similarly in terms of mean nightly abundance, mosquito species composition and nightly activity profiles. There was no statistically significant difference between indoor and outdoor HLC abundance for overall *Anopheles*. However, outdoor HLCs collected significantly more *An. vagus* than indoor HLCs, which indicates a possible exophagic preference for the species. This corroborates previous research, which has demonstrated higher *An. vagus* abundance in outdoor locations than indoor locations [27]. Furthermore, all five of the molecularly identified *Anopheles* species collected by HLCs, namely *An. aconitus*, *An. barbirostris*, *An. peditaeniatus*, *An. vagus* and *An. tessellates*, are reported as vectors of malaria in Indonesia [27–30]. The diversity of malaria vectors in Indonesia highlights the importance of continued and expanded sampling methodology. Further suggesting the likelihood of a panmictic mosquito population, there was no difference in nightly activity for indoor and outdoor collections, which mirrored each other throughout the night (Fig. 3). These similar profiles for indoor and outdoor HLCs suggest the same mosquitoes are biting inside and outside in Karama. Therefore, when behavioral and trapping profiles of outdoor and indoor species are the same, targeting indoor mosquitoes with indoor interventions would also affect the outdoor biting transmission population [31].

Traps should be selected based on the research questions being asked. This study demonstrates that net traps, particularly the KT, collect statistically viable *Anopheles* abundance rates in Karama, Indonesia. In fact, the KT performed remarkably well, either matching or statistically exceeding all other traps including HLCs for overall and species-specific abundances. Trap comparison study 1 demonstrated a statistically significantly higher mean nightly abundance for the KT compared to indoor HLCs, and suggestively higher than the outdoor HLCs (Table 2). Meanwhile, trap comparison study 2, comparing the KT, BSE, BS and outdoor HLCs found a statistical difference in overall *Anopheles* abundances between traps, though the difference was only between the KT and the BS (Table 4). Therefore, net traps collect overall *Anopheles* abundance at a consistent rate with HLCs and may be desirable as a less labor-intensive and exposure-free method for general *Anopheles* collections.

*Anopheles* species-specific differences in mean nightly abundances were examined for the KT, BSE and BS in comparison Study 2, and all performed statistically similarly to HLCs for the primary species, *An. barbirostris*, as well two secondary species, *An. barbumbrosus* and *An. umbrosus* (Table 3). Trap comparison studies 1 and 2 also demonstrated consensus for the KT collecting statistically higher abundances for *An. nigerrimus*, *An. parangensis*, *An. tessellatus* and *An. vagus* than all other traps (Table 3). Trap comparison Study 2 also found the BSE collected significantly more *An. parangensis* than HLCs.

That the KT collected the highest abundance of each species other than *An. umbrosus* compared to the BSE and BS suggests it is the optimal net sampling device in this region for the collection of *Anopheles* mosquitoes (Table 3). The KT can block mosquitoes from flying back out, whereas the BS, being a single screen, allows an intercepted mosquito to climb/fly over or around before hourly collections. The ability of KTs to prevent escaping enables them to collect a higher frequency of mosquitoes compared to the BS or BSE. Furthermore, the BS and BSE have only two sides to intercept mosquitoes. However, the KT can intercept mosquitoes on four sides which contributes to the higher proportion of mosquitoes caught compared to barrier screen sampling methods.

The KT also collected the highest species richness and scored the highest Gini-Simpsonʼs index compared to all traps in both studies. The differences in collection rates by species are most likely due to the different bionomics being targeted by each trap. The net traps target mosquito flight activity within the village, while HLCs exploit human-feeding behaviors. In other words, the high abundance and diversity of *Anopheles* mosquitoes collected with the KT compared to HLCs may indicate that the KT are a less biased collection method, as they do not specifically target anthropophagic mosquitoes like HLCs. The relatively unbiased nature of the KT makes it a powerful tool for entomological investigations.

Flight activity towards/away from the village (as measured by net traps) to biting activity (as measured by HLCs) in this study suggest that high activity in HLCs followed high activity for the KT (Figs. 3, 5). This may indicate that KTs are intercepting mosquitoes first as they enter the villages to feed, resulting in high activity in HLCs following high activity in net traps. Furthermore, *Anopheles* flying towards and away from the village had the highest activity during the early evening. This may indicate that *Anopheles* fly into the village to blood-feed and then return to rest in the surrounding forest or oviposit without resting in the village. However, further research is needed to allow for species-specific profiling of the relationship between flight times and biting times to rely less heavily on HLCs to determine biting.

The KT, BSE and BS collected blood-fed mosquitoes at statistically similar rates, between 3.0–4.0%. This suggests that these traps collect free-flying mosquitoes with either no or identical biases and that general blood-fed rates of *Anopheles* mosquitoes within the village area are 3.5–4%.

Originally, this study intended to collect *Culex* mosquitoes as well. However, after three collection days, the abundance in net traps was so high that limited resources made continued collection of culicines unfeasible (KT: *n* = 1483; BSE: *n* = 1106; BS: *n* = 463; outdoor HLCs: *n* = 216). Other research in the region reinforces the claim that net traps are useful sampling methods for *Culex* collections [7]. Nevertheless, research questions regarding *Culex* mosquitoes in the region should strongly consider a pilot study to determine the consistency of net traps in collecting this genus before implementing net traps for the sampling of *Culex* mosquitoes.

The results of this study demonstrate that the KT can provide a comprehensive evaluation of local mosquito species compositions in the region. The KT is comparable to HLCs for collecting the primary species, *An. barbirostris.* Furthermore, the KT collected statistically higher abundances for several secondary species (Table 3). Compared to HLCs they are less labor intensive: HLCs require personnel to remain awake at all hours of the night aspirating mosquitoes from their legs as they land; KTs only need to be searched every hour for mosquitoes, thus allowing multiple personnel to split the burden of collections. KTs are economical, only requiring bednet material, and are easy to take down and transport between collection sites. Furthermore, they are exposure-free, as personnel can wear repellent because they are interception traps and humans are not the main mosquito attractant. Lastly, the KT is less invasive as it does not need to be placed in local residents’ homes. The ability of the KT to intercept free-flying mosquitoes outdoors in a labor reduced, economical and exposure-free manner makes them a useful tool that should be considered when performing entomological investigations.

## Conclusions

This study demonstrates that the BS, BSE and KT methods, especially the KT method, collect overall *Anopheles* abundance and species-specific abundances at statistically similar or higher rates to HLCs in Sulawesi, Indonesia. Furthermore, the KT is exposure-free, requires less labor and does not require placement in homes. Therefore, the KT should be considered as an exposure-free alternative to HLCs for research questions regarding *Anopheles* species composition, nightly flight activity and abdominal status in this malaria endemic region.

## Data Availability

Data supporting the conclusions of this article are included within the article. Representative newly generated sequences were submitted to the GenBank database under the Accession Numbers MN203097–MN203103. The raw datasets used and/or analyzed during the present study are available from the corresponding author upon reasonable request.

## Abbreviations

BS: barrier screen
BSE: barrier screen with eaves
HLCs: human landing catches
KT: kelambu trap
